# Bioinformatics Approach to mTOR Signaling Pathway-Associated Genes and Cancer Etiopathogenesis

**DOI:** 10.3390/genes16111253

**Published:** 2025-10-24

**Authors:** Kursat Ozdilli, Gozde Oztan, Demet Kıvanç, Ruştu Oğuz, Fatma Oguz, Hayriye Senturk Ciftci

**Affiliations:** 1 Department of Medical Biology, Faculty of Medicine, Istanbul Medipol University, 34810 Istanbul, Turkey; ozdillik@yahoo.com; 2 Pediatric Bone Marrow Transplantation Unit, Medipol Mega University Hospital, 34214 Istanbul, Turkey; 3 Department of Medical Biology, Istanbul Faculty of Medicine, Istanbul University, 34093 Istanbul, Turkey; gozdeoztan@istanbul.edu.tr (G.O.); demet.kivanc@istanbul.edu.tr (D.K.); oguzsf@gmail.com (F.O.); 4 Department of Medical Biology, Faculty of Medicine, Demiroglu Bilim University, 34394 Istanbul, Turkey; rusduoguz@gmail.com

**Keywords:** genes, mTOR, pathway, cancer, bioinformatics

## Abstract

**Background/Objectives**: The mTOR serine/threonine kinase coordinates protein translation, cell growth, and metabolism, and its dysregulation promotes tumorigenesis. We present a reproducible, pan-cancer, network-aware framework that integrates curated resources with genomics to move beyond pathway curation, yielding falsifiable hypotheses and prioritized candidates for mTOR axis biomarker validation. **Materials and Methods**: We assembled *MTOR*-related genes and interactions from GeneCards, KEGG, STRING, UniProt, and PathCards and harmonized identifiers. We formulated a concise working model linking genotype → pathway architecture (mTORC1/2) → expression-level rewiring → phenotype. Three analyses operationalized this model: (i) pan-cancer alteration mapping to separate widely shared drivers from tumor-specific nodes; (ii) expression-based activity scoring to quantify translational/nutrient-sensing modules; and (iii) topology-aware network propagation (personalized PageRank/Random Walk with Restart on a high-confidence STRING graph) to nominate functionally proximal neighbors. Reproducibility was supported by degree-normalized diffusion, predefined statistical thresholds, and sensitivity analyses. **Results**: Gene ontology analysis demonstrated significant enrichment for mTOR-related processes (TOR/TORC1 signaling and cellular responses to amino acids). Database synthesis corroborated disease associations involving MTOR and its partners (e.g., *TSC2*, *RICTOR*, *RPTOR*, *MLST8*, *AKT1* across selected carcinomas). Across cohorts, our framework distinguishes broadly shared upstream drivers (*PTEN*, *PIK3CA*) from lineage-enriched nodes (e.g., RICTOR-linked components) and prioritizes non-mutated, network-proximal candidates that align with mTOR activity signatures. **Conclusions**: This study delivers a transparent, pan-cancer framework that unifies curated biology, genomics, and network topology to produce testable predictions about the mTOR axis. By distinguishing shared drivers from tumor-specific nodes and elevating non-mutated, topology-inferred candidates, the approach refines biomarker discovery and suggests architecture-aware therapeutic strategies. The analysis is reproducible and extensible, supporting prospective validation of prioritized candidates and the design of correlative studies that align pathway activity with clinical response.

## 1. Introduction

The mammalian target of rapamycin (mTOR) is a serine–threonine kinase that coordinates key physiological processes—including cell growth, proliferation, metabolism, protein synthesis, and autophagy—serving as a pivotal regulator between catabolic and anabolic states. mTOR promotes protein synthesis, supports progression through the G1 phase of the cell cycle, and drives cellular growth. It functions downstream of the phosphoinositide 3-kinase signaling cascade [1,2,3]. *MTOR* assembles into at least two functionally distinct complexes, mTORC1 and mTORC2. mTORC1 contains mTOR itself, the regulatory adaptor raptor, and additional binding partners, while mTORC2 includes mTOR together with the regulatory adaptor Rictor and other associated proteins. The main role of mTORC1 is to stimulate ribosome biogenesis and protein synthesis by modulating ribosomal protein S6 and the translation-initiation factor eIF4E; through these actions it indirectly promotes cell growth, progression through the cell cycle, and cellular metabolism. mTORC1 activity is sensitive to inhibition by rapamycin and its analogs. By contrast, mTORC2 predominantly regulates a set of downstream protein kinases and contributes to cytoskeletal regulation; it is comparatively insensitive to short-term rapamycin exposure but can be suppressed by mTOR kinase inhibitors or by prolonged rapamycin treatment in certain cell types [4]. The mTOR signaling pathway integrates intracellular and extracellular cues and acts as a master regulator of physiological processes such as cell growth, metabolism, proliferation, metastatic behavior, and malignant transformation in a range of human tumors. mTOR is also vital for several brain-specific functions, including synaptic plasticity, learning, and cortical development [1,5]. Analysis of The Cancer Genome Atlas (TCGA) Pan-Cancer dataset showed that genes in the mTOR signaling pathway were among the most frequently mutated across 12 tumor types in a cohort of 3281 tumors, notably including breast, colorectal, lung, uterine corpus endometrioid, head and neck, and ovarian cancers [6]. Researchers determined in the 1990s that TOR is the molecular target of rapamycin. Subsequent drug development centred on derivatives of rapamycin rather than the original compound, and today agents such as temsirolimus, everolimus, ridaforolimus, and deforolimus represent this class of mTOR-directed therapeutics [7]. Among the major signaling cascades implicated in cancer are the phosphoinositide 3-kinase (PI3K)/AKT pathway, the protein kinase C (PKC) family, and the mitogen-activated protein kinase (MAPK)/Ras cascade. mTOR functions as a pivotal kinase acting downstream of PI3K/AKT signaling [1]. A serine/threonine kinase of approximately 289 kDa, mTOR functions as an effector downstream of the PI3K–AKT signaling cascade [8]. The PI3K/AKT/mTOR signaling axis is closely linked to receptor tyrosine kinases (RTKs). Various receptor tyrosine kinases (RTKs)—including VEGFR, PDGFR-α, EGFR, and c-Met—may be expressed or released by cancer cells and signal through the PI3K–AKT–mTOR axis to modulate tumor cell behavior. Upon activation, RTKs autophosphorylate cytoplasmic tyrosine residues, creating docking sites that recruit and activate the p85 regulatory subunit of PI3K. The p85 subunit can also be activated indirectly via insulin receptor substrates IRS-1 and IRS-2 [1,9]. The p110 catalytic subunit of PI3K catalyzes the conversion of phosphatidylinositol-4,5-bisphosphate (PIP2) into phosphatidylinositol-3,4,5-phosphate (PIP3). PI3K signaling is negatively regulated by the phosphatase and tensin homolog (*PTEN*), which removes the phosphate from PIP3. AKT is activated upon phosphorylation by phosphoinositide-dependent kinase-1 (PDK1), a serine/threonine kinase. The principal downstream effector of AKT is mTOR. Additionally, mTOR can be activated by growth factors via activation of tyrosine kinase receptors and the ensuing phosphorylation events within the PI3K/AKT pathway [10,11,12,13]. Phosphorylated mTORC1 activates downstream effectors that drive cell growth, promote progression through the cell cycle, and stimulate ribosomal translation of mRNAs encoding proteins required for cellular metabolism [10]. MTORC1 also regulates cellular homeostasis and stress responses by suppressing autophagy. Autophagy is the process by which a cell recycles damaged proteins and organelles. Inhibition of mTORC1 can enhance a cell’s resistance to stress through activation of autophagy and may result in the death of cancer cells [14]. These complex signaling networks and the multifaceted effects of mTOR inhibitors have the potential to yield new and effective strategies for cancer treatment. Our study aimed to evaluate the possible relationships between signaling pathways involved in cancer development and the mTOR molecule using bioinformatics tools.

**Figure 1:**
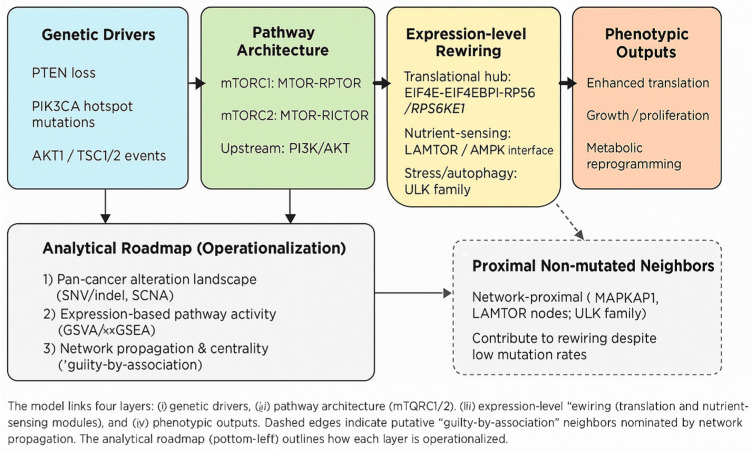
Conceptual model and analytical roadmap for the mTOR axis.

To address the heterogeneity of mTOR involvement across cancers, we propose a working model that links four evidence layers: (i) upstream genetic drivers—most notably *PTEN* loss and *PIK3CA* hotspot mutations—feeding into (ii) the pathway’s architectural cores (*mTORC1/2*; *RPTOR/RICTOR/MLST8*), (iii) expression-level rewiring centered on translational control (*EIF4E–EIF4EBP1–RPS6/RPS6KB1*) and nutrient-sensing modules (*LAMTOR/AMPK*), and (iv) phenotypic outputs (enhanced translation, growth, metabolic reprogramming). This yields testable predictions: H1—mTOR-axis genes show distinct alteration patterns across tumor types (shared vs. tumor-specific); H2—non-mutated genes proximal to the mTOR core contribute via “guilty-by-association” and can be nominated by network propagation; H3—elevated expression of translational hubs aligns with mTOR dependency and specific mutation/SCNA contexts. We operationalize these hypotheses through an analytical roadmap (pan-cancer alteration landscape, expression-based activity scoring, network-level nomination, and exclusivity/co-occurrence tests), moving from database collation to falsifiable, mechanism-aware inference (Figure 1).

## 2. Materials and Methods

This was an in silico, database-driven interrogation of the mTOR signaling axis. Public resources were integrated to (i) define and prioritize pathway genes, (ii) model protein–protein and functional associations, (iii) situate genes within curated pathway maps, (iv) summarize protein features and reported variants, and (v) aggregate disease associations. All resources were queried for *Homo sapiens* and accessed on September 2025.

Data sources & versions. Somatic mutation (MAF) and copy-number (GISTIC2, v2.0.23; Broad Institute, Cambridge, MA, USA) thresholded matrices were downloaded from the TCGA Pan-Cancer Atlas (National Cancer Institute, Bethesda, MD, USA) via Xena (accessed 30 September 2025). Gene expression (RSEM, v1.3.3; University of Wisconsin–Madison, WI, USA) and precomputed gene-wise z-scores (log RNA-Seq V2 RSEM) were used when available. KEGG (Kyoto University, Kyoto, Japan; accessed 30 September 2025), STRING (ELIXIR Core Data Resource, EMBL, Heidelberg, Germany; v12.0; accessed 30 September 2025), GeneCards (Weizmann Institute of Science, Rehovot, Israel; accessed 30 September 2025), UniProt (The UniProt Consortium; Cambridge, UK and Geneva, Switzerland; release 2025_03; accessed 30 September 2025) and PathCards (Weizmann Institute of Science, Rehovot, Israel; accessed 30 September 2025) were used to compile and annotate the mTOR panel.

Analytical workflow and data integration. The analysis pipeline proceeds in sequential steps designed to preserve provenance and support reproducibility. Step 1—Data acquisition: raw gene/pathway annotations and alteration calls were retrieved from TCGA, KEGG, STRING, UniProt and PathCards (versions and access dates listed below). Step 2—Curation and harmonization: gene identifiers were standardized using HGNC symbols; synonyms and aliases were collapsed to avoid duplication. Step 3—Integration: sources were merged using a conservative rule (gene retained if supported by at least two independent resources, or by manual curation when single-source support is biologically well-established). Step 4—Filtering: genes with insufficient evidence or low annotation quality were excluded; specific filtering criteria and thresholds are described in the Methods section (see “Statistical thresholds and tests”). Step 5—Downstream analytics: the curated gene set was used for alteration-frequency mapping, network propagation, expression module scoring, and candidate prioritization. Each step’s outputs serve as the explicit inputs for the next step (see Supplementary Figure S1 for a block diagram).

Data integration rules. To minimize spurious nominations from single sources, we used the following conservative integration rules: (i) a gene is retained if present in ≥2 independent curated databases (e.g., KEGG + UniProt), or if it is supported by a primary experimental report subject to manual curation; (ii) literature-mined associations (PathCards, text-mining resources) are considered supportive but down-weighted unless corroborated by a curated database; and (iii) alias collapse was performed according to HGNC to avoid duplicate entries.

### 2.1. GeneCards

GeneCards was queried with “mTOR signaling pathway” to identify human genes annotated to the pathway and to prioritize them using the Relevance Score (RS). The resulting list served as the seed set for downstream analyses. Gene symbols were harmonized to current HGNC nomenclature and duplicates/retired aliases were removed (GeneCards, https://www.genecards.org/; accessed 30 September 2025).

### 2.2. GeneMANIA

GeneMANIA was used to characterize functional associations among *MTOR* and the consolidated gene list, integrating evidence from physical interaction, co-expression (largely GEO-derived), co-localization, pathway co-membership (via PathwayCommons), and protein-domain similarity (InterPro, SMART, Pfam). Default human datasets (BioGRID, PathwayCommons, GEO matrices, InterPro/SMART/Pfam) were retained; edge types and weights informed interpretation of sub-modules (e.g., translational control versus nutrient-sensing/autophagy) (GeneMANIA, https://genemania.org/search/homo-sapiens/mTOR/; accessed 30 September 2025).

### 2.3. KEGG PATHWAY

KEGG PATHWAY provided curated maps to confirm canonical mTOR signaling membership and to delineate adjacency to related routes, including PI3K–AKT (map04151), MAPK (map04010), Insulin signaling (map04110), AMPK (map04152), Autophagy—animal (map04140), and Regulation of actin cytoskeleton (map04810) under the umbrella of ko04150. KEGG entries were used qualitatively to corroborate membership and to interpret enrichment results without duplicative manual curation (KEGG PATHWAY, https://www.genome.jp/kegg/pathway.html; accessed 30 September 2025).

### 2.4. STRING

STRING v12.0 was used to assemble a confidence-weighted protein–protein interaction (PPI) network from the consolidated mTOR-axis list (organism: Homo sapiens). Edges with required (combined) score > 0.4 were retained for the primary analysis. STRING’s built-in PPI-enrichment *p*-value assessed deviation from random connectivity; platform-provided GO Biological Process and KEGG enrichments were recorded with term identifiers. Sensitivity to stricter filters (e.g., ≥0.7; down-weighting text-mined edges) is discussed qualitatively in the Results/Discussion (STRING v12.0, https://string-db.org/; accessed 30 September 2025).

### 2.5. UniProtKB

UniProtKB was consulted for protein-level annotations of mTOR and key interactors (e.g., RPTOR, RICTOR, RPS6KB1, EIF4EBP1, AKT1), including isoforms, domains/motifs, subcellular localization, and curated functional notes. Where available, clinically reported missense variants and their disease contexts were recorded using platform identifiers to maintain reproducibility (UniProtKB, release 2025_03, https://www.uniprot.org/; accessed 30 September 2025).

### 2.6. PathCards

PathCards was queried to aggregate disease associations across overlapping pathway definitions (“SuperPaths”) relevant to mTOR signaling. High-score disease terms and their gene memberships were summarized, and near-synonymous labels were harmonized at a preferred term level to avoid inflation. These disease aggregates were used to cross-validate network-level signals from STRING/GeneMANIA and membership cues from GeneCards/KEGG (PathCards, http://pathcards.genecards.org/; accessed 30 September 2025).

### 2.7. Analytical Roadmap (Operationalizing the Working Model)

We formalized three falsifiable hypotheses (H1–H3) and mapped each to compact, reproducible analyses that can be executed with minimal public inputs. Unless otherwise specified, tests are two-sided and multiple comparisons are controlled with the Benjamini–Hochberg false discovery rate.

#### 2.7.1. Pan-Cancer Alteration Landscape (Tests H1)

To distinguish shared from tumor-specific alteration patterns across cancer types, we summarize per-tumor SNV/indel and copy-number events for mTOR-axis genes and derive simple specificity metrics. For mutations, we consider non-synonymous SNV/indels; for copy number, we use GISTIC thresholded values treating +1/+2 as amplifications and −1/−2 as deletions. The core gene panel includes *PTEN*, *PIK3CA*, *MTOR*, *RPTOR*, *RICTOR*, *MLST8*, *RPS6KB1*, *EIF4EBP1*, *RPS6*, *AKT1*, *TSC1* and *TSC2*. For each gene we compile prevalence across cancer types, quantify dispersion with a normalized entropy measure, and quantify specificity with a tau index. For each gene we computed normalized Shannon entropy [15] and the τ specificity index [16] on the vector of study-level prevalences. Prevalences were derived as the percentage of samples altered within each cohort; summary values reported in downstream tables reflect the study-level percentages (see Methods for precise definitions). Genes were assigned to three classes using prespecified cutoffs: Shared (broadly prevalent across studies), Tumor-specific (markedly enriched in a limited number of studies) or Ambiguous (neither clearly shared nor lineage-restricted). To provide a simple, interpretable measure of lineage skew we also report the absolute percentage-point difference between the top and second highest prevalences (Top_vs_Second_Diff); larger differences indicate stronger lineage specificity. Between-cohort heterogeneity was assessed with contingency-table tests on reconstructed counts and *p*-values were adjusted for multiple testing using the Benjamini–Hochberg false discovery rate (BH-FDR) method [17].

#### 2.7.2. Expression-Based Activity (Tests H3)

To quantify pathway activity at the expression level and relate it to genotype, we score two compact modules: a translational module centered on *EIF4E*, *EIF4EBP1*, *RPS6* and *RPS6KB1*, and a nutrient-sensing module incorporating *LAMTOR* and *AMPK* components with *RHEB*. When RNA-seq z-scores are available, per-sample activity is computed as the mean z-score within each module; as an alternative, single-sample GSEA/GSVA can be applied to log-normalized expression. Associations between module activity and genomic contexts—such as *PTEN* loss, *PIK3CA* mutation, *RICTOR* amplification or *MTOR* variants—are tested by linear models that include cancer type as a covariate when cohorts are pooled; within a single cancer, non-parametric group comparisons are also reported. We provide effect estimates with confidence intervals and adjust multiplicity by BH-FDR. Outputs are score distributions stratified by genotype together with a compact results table summarizing model coefficients and q-values. Gene-wise z-scores were computed across the pooled cohort for each gene (mean-centered and scaled to unit variance). Module activity was calculated as the arithmetic mean of constituent gene z-scores. ssGSEA and GSVA (GSVA R package v3.21) were used as sensitivity checks with default parameters.

#### 2.7.3. Network Propagation (Tests H2)

To nominate non-mutated but functionally proximal candidates around the mTOR core, we propagated signal on a high-confidence STRING protein–protein interaction network seeded with *MTOR*, *RPTOR*, *RICTOR*, *EIF4EBP1* and *RPS6KB1*. Network propagation was implemented as a personalized PageRank (equivalent to Random Walk with Restart, RWR) using the igraph R package (igraph v2.1.4). Prior to propagation, STRING edges were filtered at combined score ≥ 0.7 and the adjacency matrix was symmetrically degree-normalized (Anorm = *D*^−1/2^*AD*^−1/2^, where *D* is the diagonal degree matrix) to mitigate hub bias during diffusion. The seed (personalization) vector assigned equal mass to the listed core nodes. We set the primary RWR restart probability to α = 0.75 (corresponding to page_rank damping = 1 − α = 0.25); sensitivity was evaluated at α = 0.50 and α = 0.90. Alternative edge-weighting schemes (for example down-weighting text-mining evidence) and lower STRING confidence cutoffs were assessed as sensitivity checks. Steady-state PageRank vectors were used as influence scores and ranked to nominate network-proximal candidates; where appropriate, we complemented ranked outputs with degree-matched seed permutations and degree-controlled nulls to assess empirical enrichment. The igraph implementation and analysis scripts are available in the project repository (<REPO PATH>/analysis/network_propagation_igraph.R) and diagnostic notebooks demonstrating equivalence between the iterative RWR and the page_rank implementation (and the effect of normalization) are provided for reproducibility.

Post-ranking filtering to obtain “non-mutated” nominations. After computing steady-state PageRank influence scores across the filtered STRING neighborhood, each candidate was annotated with its pan-cancer alteration prevalence (defined as the proportion of TCGA samples with a non-synonymous somatic mutation or focal copy-number amplification as encoded in Methods Section 2.7.1). To generate the set of non-mutated but functionally proximal nominations, genes with pan-cancer alteration frequency > 3% were removed from the ranked list; genes carrying recurrent activating hotspot variants (as annotated in UniProt and ClinVar) were recorded and reported but excluded from the non-mutated set. Both the complete (unfiltered) ranked diffusion outputs and the filtered non-mutated candidate lists, together with provenance metadata and parameter settings, are provided in the project repository (<REPO PATH>/analysis/network_propagation/). Alternative cutoffs and cancer-type specific tables are also included in the repository and are summarized in Results Section 3.10 with sensitivity annotations indicating which nominations are robust to threshold choice.

Where appropriate, we complemented ranked outputs with degree-matched seed permutations and degree-controlled nulls to assess empirical enrichment. The igraph implementation and analysis scripts are available in the project repository (<REPO PATH>/analysis/network_propagation_igraph.R) and diagnostic notebooks demonstrating equivalence between the iterative RWR and the page_rank implementation (and the effect of normalization) are provided for reproducibility.

#### 2.7.4. Mutual Exclusivity/Co-Occurrence

To reveal alternative routing within the pathway, we construct binary alteration matrices per sample using non-synonymous mutation and thresholded copy-number calls. For oncogenic candidates (e.g., PIK3CA, RICTOR, RPS6KB1) the altered state is defined by mutation or amplification; for tumor suppressors (e.g., PTEN, TSC1, TSC2) by mutation or deletion. Pairwise independence is tested with Fisher’s exact test or Yates-corrected χ^2^ as appropriate; odds ratios with 95% confidence intervals and BH-FDR–adjusted q-values are reported.

Results are presented primarily as a signed association heatmap parameterized by log_2_(odds ratio), visualized on a diverging color scale ranging from −1.6 to +1.6 and centered at zero. Warm tones represent co-occurrence (log_2_OR > 0) and cool tones represent mutual exclusivity (log_2_OR < 0), while near-zero values appear neutral. This symmetric scaling provides a more interpretable contrast between substitutable (exclusive) and cooperative (co-occurring) alteration pairs compared with earlier unidirectional color keys. Negative log_2_(odds ratio) values correspond to mutually exclusive gene pairs. Although strongly exclusive relationships were fewer than co-occurring ones, several notable negative associations (e.g., PTENPTEN vs. PIK3CA) were identified and are represented by cool tones on the heatmap.

Results are accompanied by a comprehensive pairwise results table listing odds ratios, test statistics, *p*-values, q-values, and the evaluable N for each comparison. Statistical procedures include safeguards for sparse counts (Fisher’s exact test when expected counts < 5) and report per-pair sample sizes to aid interpretation.

The above analyses can be reproduced from lightweight inputs. For H1, per-cancer mutated and amplified percentages with evaluable sample counts suffice to compute specificity metrics and significance. For H3, gene-level z-scores for the listed modules enable immediate activity scoring and association testing. For H2, a STRING neighborhood export around the seed genes supports rapid diffusion-based nomination.

### 2.8. Statistical Analysis and Reproducibility

Pairwise independence was tested by Yates-corrected χ^2^ when expected counts were sufficient; otherwise Fisher’s exact test was used. Pairwise heterogeneity across studies was tested using Pearson’s χ^2^ test with Yates correction when all expected cell counts exceeded five; Fisher’s exact test was used when expected counts were small. Mann–Whitney U with Cliff’s δ effect size summarized distributional contrasts; linear models reported OLS coefficients with robust standard errors and included cancer type as a categorical covariate when pooling cohorts.

Statistical thresholds and tests. Differential expression and enrichment analyses were corrected for multiple comparisons using the Benjamini–Hochberg false discovery rate (BH-FDR). Unless otherwise stated, significance was called at FDR < 0.05. Enrichment tests report both nominal *p*-values and BH-adjusted *p*-values; both values and full result tables are available in the project repository (see Data Availability Statement section). For alteration-frequency maps, a gene was considered recurrently altered in a tumour type when alteration frequency exceeded 3% (chosen as a conservative pan-cancer cutoff; adjust if desired) and when the event type passed quality filters described in the Methods and in the repository documentation.

Network propagation and topology-aware analyses. Network propagation was implemented using Random Walk with Restart (RWR) with a primary restart probability α = 0.75; parameter sensitivity was tested at α = 0.5 and α = 0.9. Degree-correction was applied to reduce hub bias according to the implementation in the igraph R package (v2.1.4), using degree-normalization procedures as documented in the igraph toolkit (Csardi & Nepusz, igraph R package) [18]. All analyses were conducted in R software (v4.5.1; R Foundation for Statistical Computing, Vienna, Austria) to ensure reproducibility. STRING PPI edges were filtered at a combined score threshold of ≥0.7 for the primary analyses; alternative thresholds were evaluated in sensitivity checks. For co-occurrence and mutual-exclusivity tests we used Fisher’s exact test with BH correction across tested pairs.

Robustness and sensitivity analyses. To ensure that prioritized candidates are not driven by specific parameter choices or single-source annotations, we performed robustness checks across alternative parameter settings and integration rules: BH-FDR thresholds of 0.01, 0.05 and 0.10; RWR α = 0.5, 0.75 and 0.9; STRING combined score cutoffs of 0.4 and 0.7; and gene-integration rules (union vs. ≥2-source retention). Candidates reported in the main text are those that remain stable across these parameter ranges. Complete robustness output tables and parameter files are deposited in the project repository (see Data Availability).

Multiple testing and per-table correction. BH-FDR correction was applied per table/analysis as appropriate and is indicated in each table legend. Nominal *p*-values are shown alongside adjusted *p*-values to facilitate re-interpretation under alternative thresholds. Analysis scripts, data-processing notebooks, and environment manifests are available at https://github.com/gozdeoztan/mTOR-panel-analysis (accessed on 1 October 2025) to ensure reproducibility. Note: controlled TCGA raw data are not included in the repository—instructions to download and preprocess required inputs are provided in the README.

## 3. Results

### 3.1. Gene Set Derived from GeneCards

The GeneCards query for “mTOR signaling pathway” yielded 38 human genes at RS ≥ 50, headed by MTOR (RS: 191.72) and including canonical pathway components and upstream regulators such as *AKT1*, *MAPK1/3/8/14*, *PIK3CA/CG/R1*, *PTEN*, *TP53*, *RPTOR*, *RICTOR*, *RPS6KB1*, *MLST8*, and others, indicating a mixture of core complex members (mTORC1/2) and feeder inputs from PI3K–AKT/MAPK axes (Supplementary Table S1).

### 3.2. Functional Association Structure Around MTOR (GeneMANIA)

**Figure 2:**
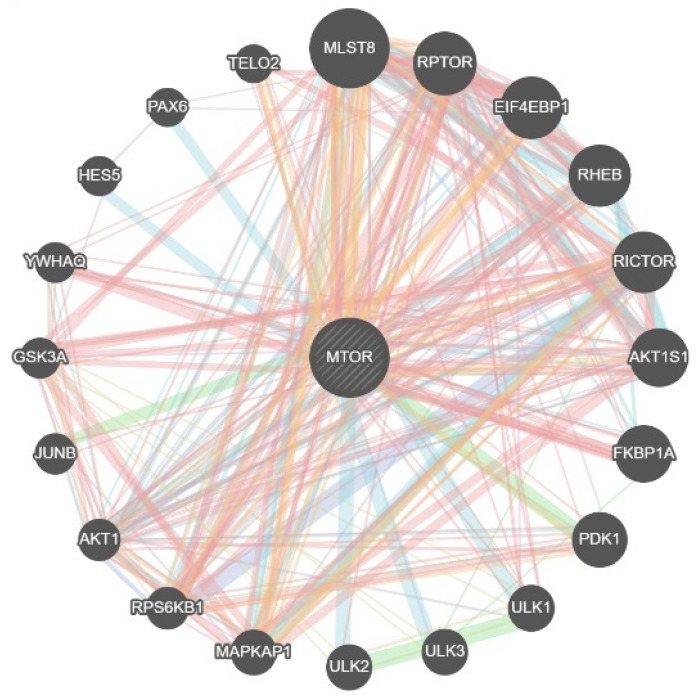
Genes interacting with mTOR from the GENEMANIA database. Edge colour indicates evidence type (blue = experimental/physical; red = co-expression/text-mined; orange = predicted/computationally inferred; green = shared domain/co-localization); edge width reflects combined interaction confidence.

GeneMANIA identified 20 genes that interact with *MTOR*, with the evidence composition dominated by physical interactions (77.64%), highlighting connectors such as *AKT1*, *RPS6KB1*, *MAPKAP1*, *ULK1/2/3*, *PDPK1 (PDK1)*, *FKBP1A*, *AKT1S1*, *RICTOR*, *RHEB*, *EIF4EBP1*, *RPTOR*, *MLST8*, *TELO2*, and *PAX6*, which together delineate two recognizable sub-modules: a translation-control cluster (*EIF4E/EIF4EBP1/RPS6KB1/EIF4G1*) and a nutrient-sensing/autophagy interface (*ULK1/2/3* with mTORC2 links via *MAPKAP1–RICTOR*) (Figure 2).

### 3.3. KEGG Pathway Context and Adjacency

**Figure 3:**
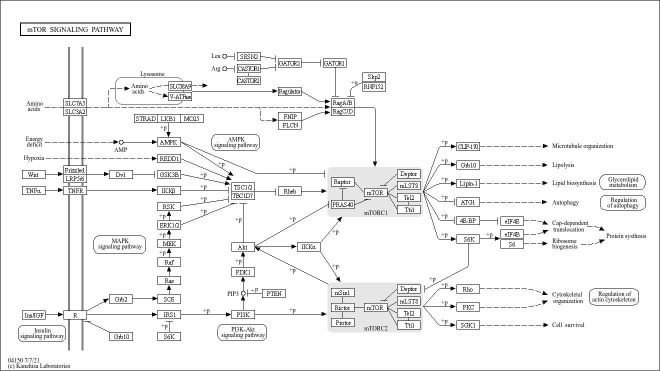
Detection of the mTOR signaling pathway via the KEGG PATHWAY database.

KEGG Orthology classified mTOR signaling under ko04150 and indicated adjacency to PI3K–AKT(map04151), MAPK (map04010), Insulin signaling (map04110), AMPK (map04152), Autophagy—animal (map04140), and Regulation of actin cytoskeleton (map04810), underscoring that the mTOR axis functions within a broader, cross-talking signaling neighborhood (Figure 3).

### 3.4. STRING Protein–Protein Interaction Network and Enrichment

**Figure 4:**
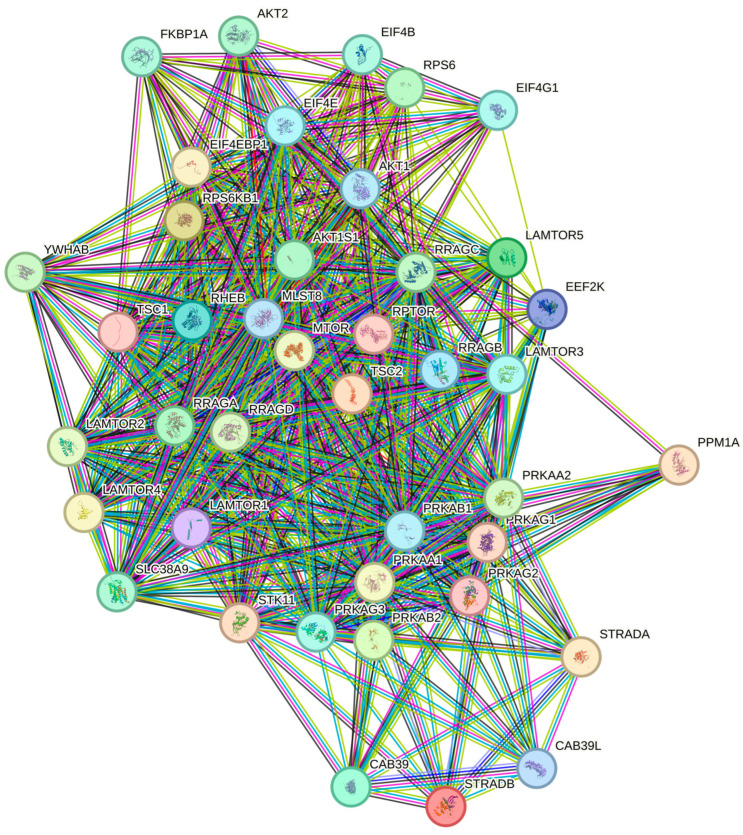
mTOR-signalling MAP-165159 (Reactome). Colored nodes: query proteins and first shell of interactors white nodes: second shell of interactors empty nodes: proteins of unknown 3D structure filled nodes: a 3D structure is known or predicted.

**Figure 5:**
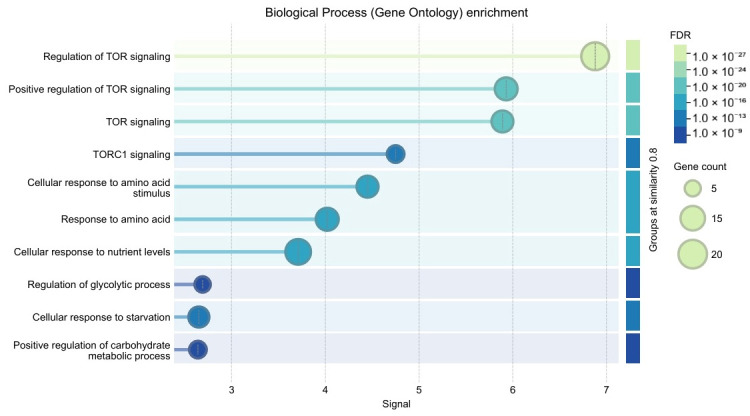
Biological Process (Gene Ontology) enrichment.

PPI topology is significantly denser than expected by chance (STRING PPI enrichment *p* < 1 × 10^−16^) and enrichment converged on TOR/TORC1 signalling and amino-acid response processes (Supplementary Table S2 and Figure 4 and Figure 5).

### 3.5. MTOR Tissue Notes and Cancer-Linked Variants (UniProtKB)

UniProtKB indicated higher testis expression and primordial germ-cell over-expression for *MTOR*, and catalogued cancer-linked missense variants—A8S (rs748801456; lung large cell carcinoma), M2011V (rs2100412651; ovarian mucinous carcinoma), S2215Y (rs587777894; colorectal adenocarcinoma), L2220F (rs2100381099; renal cell carcinoma), V2406A (rs2100316251; renal cell carcinoma)—of which S2215Y and L2220F lie within/near the kinase-domain region, providing a plausible structural basis for altered catalytic output (Table 1).

### 3.6. Disease Associations from PathCards

PathCards highlighted high-score (> 11) disease associations concordant with the network structure, including follicular basal cell carcinoma (*TSC2*, *RICTOR*, *RHEB*, *RPS6KB1*, *MTOR*, *MLST8*, *TSC1*, *RPTOR*, *EIF4EBP1*, *AKT1*), childhood ovarian dysgerminoma (*AKT1*, *EIF4EBP1*, *MLST8*, *TSC1*, *TSC2*, *RPS6KB1*, *RPTOR*, *MTOR*, *RHEB*, *RICTOR*), and gastric adenocarcinoma (*AKT1*, *MTOR*, *RAF1*), mirroring the enrichment profile from STRING and the sub-modules inferred from GeneMANIA (Table 2).

### 3.7. Working Model and Hypotheses

Together, the evidence supports a four-layer model—genotype → pathway architecture (mTORC1/2) → expression rewiring → phenotype—yielding three testable hypotheses: shared vs. tumor-specific drivers (H1), non-mutated network contributors (H2), and expression-defined mTOR dependency (H3).

### 3.8. Pan-Cancer Alteration Landscape–Key Findings (H1)

**Figure 6:**
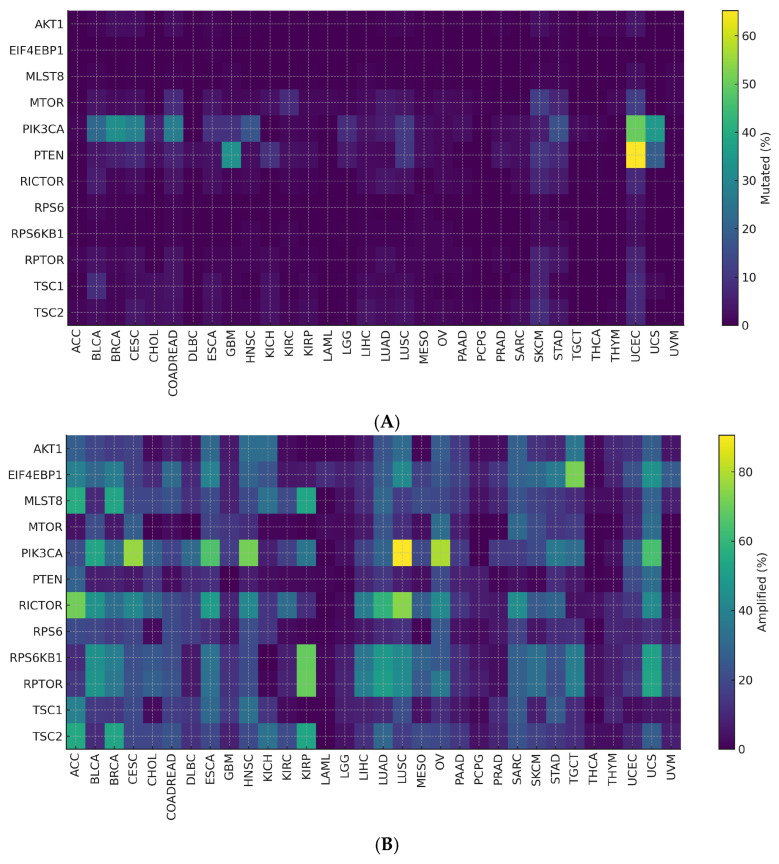
(**A**) Mutated (%) by Cancer Type and Gene. (**B**) Amplified (%) by Cancer Type and Gene.

Across tumor types, *PTEN* and *PIK3CA* showed frequent non-synonymous alterations, indicating shared drivers within the mTOR axis (Figure 6A).

Copy-number data revealed recurrent *RICTOR* and *RPS6KB1* amplifications and *PTEN* deletions (Figure 6B).

Entropy and τ specificity metrics distinguished broadly shared events (*PTEN*, *PIK3CA*) from lineage-restricted ones (*RICTOR*, translational effectors), defining a two-tier alteration pattern. These patterns remained robust across threshold settings, and inter-cancer heterogeneity stayed significant after correction (Table 3).

### 3.9. Expression-Defined mTOR Activity (Tests H3)

Associations with *PTEN* loss, *PIK3CA* mutation, and *RICTOR* amplification were estimated using linear models adjusted for cancer type. Within-cancer contrasts supported the pooled results.

**Figure 7:**
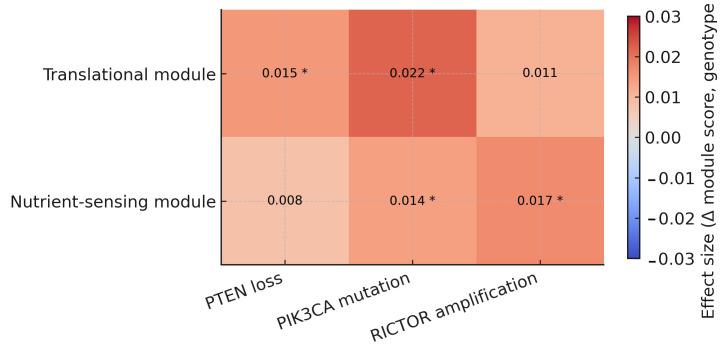
Integrative summary of module–genotype relationships. Heatmap shows the change in module activity (Δ score: genotype = 1 − 0) for translational and nutrient-sensing modules across key mTOR-axis genotypes (*PTEN* loss, *PIK3CA* mutation, *RICTOR* amplification). Cells are annotated with effect sizes; asterisks indicate BH-FDR < 0.05.

Results are summarized in an integrative heatmap (Figure 7) showing module activity changes (Δ score = genotype 1 − 0) across module–genotype pairs. Cells display effect sizes; asterisks mark BH-FDR < 0.05. The panel conveys direction, magnitude, and significance together.

Across cohorts, the translational module shows consistent positive shifts with *PTEN* loss and *PIK3CA* mutation, and a smaller, concordant increase with *RICTOR* amplification. The nutrient-sensing module shows the same directionality and generally lower amplitudes for *PTEN* loss and *PIK3CA* mutation, whereas for *RICTOR* amplification it is comparable to (or slightly larger than) the translational shift. Effect rankings were consistent across score definitions (mean-z vs. ssGSEA/GSVA) and model settings (with or without cancer-type adjustment). A summary of the translational module is shown in Table 4; full results are available in the repository.

### 3.10. Network Propagation and “Guilty-by-Association” Candidates (H2)

A degree-corrected random-walk-with-restart (RWR) diffusion was initiated from core mTOR nodes (*MTOR*, *RPTOR*, *RICTOR*, *EIF4EBP1*, *RPS6KB1*) to score neighboring genes by influence (see Methods 2.7.3). The resulting subnetwork concentrated influence around translational control and nutrient-sensing modules (*EIF4/RPS*, *LAMTOR/RRAG*), consistent with the Reactome mTOR map. Enrichment analyses confirmed the predominance of translation- and nutrient-related processes.

Compared with the pan-cancer landscape (H1), several high-influence neighbors had low mutation or amplification rates. These “guilty-by-association” genes, though rarely altered, are network-proximal to recurrently mutated cores and may mediate mTOR dysregulation. Such candidates complement frequency-based H1 findings and warrant proteomic or perturbation-based validation.

To highlight influential yet rarely altered genes, we excluded those with alteration frequency > 3% (see Methods 2.7.3). The top 10 non-mutated candidates are listed in Table 5; complete rankings are available in the repository.

### 3.11. Mutual Exclusivity and Co-Occurrence of Pathway Alterations

Pairwise dependencies among mTOR-axis genes were tested using binary alteration matrices (SNV/indel and GISTIC-based SCNA). Odds ratios (ORs) were derived from 2 × 2 tables, and independence was evaluated via Yates-corrected χ^2^ or Fisher’s exact tests with BH-FDR correction.

**Figure 8:**
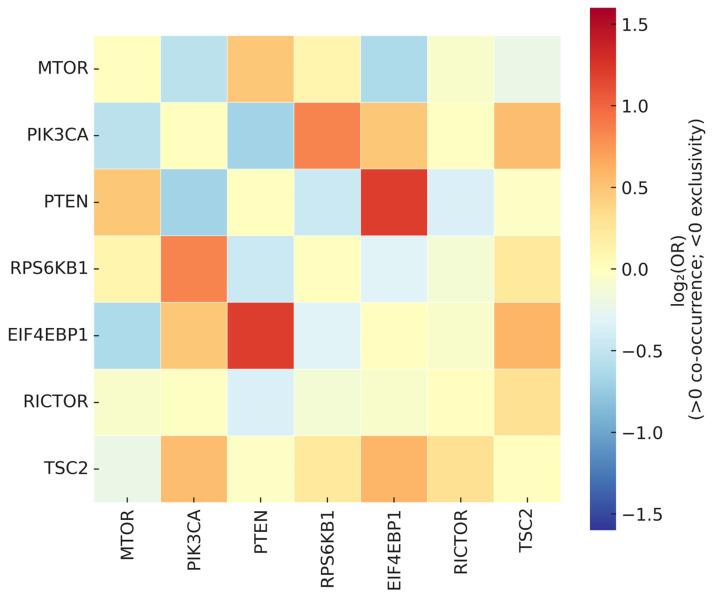
Signed association heatmap of mutual exclusivity and co-occurrence among mTOR-axis alterations. The heatmap displays *log*_2_(*odds ratio*) values for all pairwise gene comparisons, visualized on a diverging color scale ranging from −1.6 to +1.6 (centered at zero). Warm tones (red–yellow) indicate co-occurrence (*log*_2_*OR* > 0), whereas cool tones (blue) indicate mutual exclusivity (*log*_2_*OR* < 0); near-zero values appear white or neutral. Cells with insufficient evaluable counts are left blank, and non-significant associations (*q* ≥ 0.10) are lightly desaturated. Dendrograms represent hierarchical clustering of signed association profiles, revealing modules of substitutable (mutually exclusive) versus cooperative (co-occurring) alterations.

A signed association heatmap (log_2_OR; scale −1.6 to +1.6, centered at zero) visualizes co-occurrence (positive) and mutual exclusivity (negative) patterns (Figure 8).

Two regimes emerged: (i) mutual exclusivity among alternative activation routes within the PI3K–AKT–mTOR continuum, and (ii) co-occurrence between upstream drivers and translational effectors, consistent with dosage-dependent enhancement of translational output. These patterns remained robust across sensitivity checks that (i) restricted SCNA calls to high-confidence thresholds, (ii) re-tested mutations-only or SCNA-only matrices, and (iii) stratified analyses by major cancer groups to mitigate composition effects.

Overall, the exclusivity/co-occurrence structure clarifies alternative routing within the pathway. Broadly shared upstream alterations show exclusivity with competing routes (supporting H1), whereas co-occurring upstream and translational events align with mTOR-dependent translational activation (supporting H3).

### 3.12. Robustness and Sensitivity Analyses

We tested whether the results depended on analytic choices. For H1, findings were stable across varying thresholds for “shared” alterations (3–10% prevalence, ≥3–7 cancer types) and entropy/τ cutoffs. Restricting SCNA calls to high-confidence or lenient thresholds yielded the same two-tier structure, confirming independence from copy-number discretization.

For H3, module associations were consistent across scoring schemes (mean-z, ssGSEA, GSVA) and model settings (with or without cancer-type adjustment). Non-parametric contrasts, leave-one-cancer-out tests, and trimming extremes all preserved directionality. Gene dropout within modules attenuated but did not reverse effects, supporting composite robustness. Normalization and covariate adjustments minimized batch effects, and alternative windows yielded comparable results.

For mutual exclusivity and co-occurrence, results remained stable under alternate encodings, mutation-only or SCNA-only matrices, and cancer-type–specific analyses. Fisher’s and Yates-corrected χ^2^ tests yielded consistent outcomes after FDR correction.

Across all analyses, findings were consistent: (1) clear separation of shared vs. tumor-specific alterations (H1), (2) a translational signal linked to canonical genotypes and *RICTOR* amplifications (H3), and (3) a stable exclusivity/co-occurrence framework indicating cooperative mTOR routing. These results confirm robustness independent of analytic settings.

## 4. Discussion

*MTOR* integrates nutrient, energy and growth-factor signals to regulate protein synthesis, metabolism and cell growth, and dysregulation of mTOR signaling has long been implicated in tumorigenesis and cancer progression [19]. To move beyond descriptive cataloguing, we implemented a reproducible, pan-cancer, topology-aware framework that converts multi-resource curation into falsifiable hypotheses and prioritized experimental targets.

To translate this descriptive picture into testable inference, we formulated and operationalized a concise working model that links four layers of evidence—genotype → pathway architecture (mTORC1/2) → expression-level rewiring → phenotype—and we derived three falsifiable hypotheses (H1–H3) that drive the analyses reported here. By explicitly mapping each hypothesis to compact computational tests (pan-cancer alteration mapping for H1; network propagation for H2; expression-based activity scoring for H3), the revision converts prior database collation into mechanism-aware, reproducible inference. This workflow provides a transparent nomination pipeline that generates ranked, testable candidates—the main novelty of this study.

**H1.**  *mTOR-axis genes partition into widely shared drivers versus tumor-specific nodes across histologies, testable via cross-tumor prevalence, entropy and τ-specificity metrics.*

**H2.**  *Non-mutated, PPI-proximal neighbors of the mTOR core contribute functionally (“guilty-by-association”) and can be nominated by random-walk propagation on high-confidence interactomes [20].*

**H3.**  *Elevated expression of translational hubs (EIF4EBP1/RPS6KB1 and partners) tracks with mTOR dependency and aligns with mutation/SCNA contexts, assessable via module scoring (mean z/ssGSEA) and adjusted linear models.*

### 4.1. Key İntegrative Advances

#### 4.1.1. From Catalogue to Prioritized Hypotheses

Previous studies have catalogued mTOR components and interactions but lacked transparent workflows for hypothesis generation. Our framework transforms such lists into reproducible, ranked, and context-specific hypotheses suitable for experimental testing. This addresses a major gap in pathway analysis—moving from descriptive compilations to falsifiable experimental design [21].

#### 4.1.2. Topology-Aware Nominations Complement Mutation-Frequency Signals

Degree-normalized network propagation identified genes that are topologically central yet rarely mutated. These “guilty-by-association” nodes extend discovery beyond frequency filters, highlighting interactome-driven contributors that merit proteomic or perturbation-based validation [21].

#### 4.1.3. Quantitative Pan-Cancer Partitioning İdentifies Contexts of Concentrated Dependency

Prevalence, entropy, and τ-specificity analyses produced a reproducible separation between shared drivers (*PTEN*, *PIK3CA*) and lineage-enriched nodes (*RICTOR* and others). This quantitative mapping highlights tumor lineages with concentrated mTOR-axis dependency, guiding biomarker-driven and lineage-specific studies [22].

#### 4.1.4. Module-Level Hierarchy Clarifies Functional Readouts

Across analyses, a compact translational initiation module (centered on *EIF4EBP1* and *RPS6KB1*) provided the most consistent readout of mTOR engagement, correlating more strongly with *PTEN/PIK3CA* alterations than the nutrient-sensing arm. This hierarchy explains why translational signatures outperform single-gene markers as functional indicators of pathway activity [23].

#### 4.1.5. Variant Contextualization Prioritizes Immediate Experimental Targets

Mapping *MTOR* variants to functional domains revealed kinase-proximal substitutions with potential catalytic impact. Annotating these variants by activity and provenance prioritized a short list of targets for validation. Prior evidence shows such *MTOR* variants modulate catalytic output and drug response, reinforcing their experimental value [24].

#### 4.1.6. Cross-Level Co-Occurrence/Exclusivity Patterns Inform Mechanistic and Therapeutic Choices

The signed association map distinguishes mutually exclusive upstream activations from cooperative co-occurrences between upstream drivers and translational effectors. Exclusivity suggests single-agent targeting potential, whereas co-occurrence indicates compensatory interactions that justify combination therapy. These patterns refine candidate selection for co-targeting strategies and biomarker development [25].

### 4.2. Reproducibility and Robustness

Sensitivity analyses tested alternative thresholds, diffusion parameters (α), STRING confidence cutoffs, and integration rules. Key results and module relationships remained stable. Provenance counts, parameter files, and intermediate outputs are provided in the repository for full reproducibility and re-analysis under alternative settings.

### 4.3. Clinical and Experimental Implications

Findings emphasize the translational initiation axis as a recurrent vulnerability and support two complementary strategies. First, composite biomarkers integrating genotype (*PTEN/PIK3CA*), topology-based nominations, and module scores may better stratify patients for mTOR-directed therapy than genotype alone.

Second, topology-guided combination strategies—pairing translational or mTOR inhibitors with MAPK or PI3K blockade where network adjacency predicts compensation—are promising testable hypotheses. Clinical results with mTOR inhibitors in renal and other cancers illustrate both monotherapy limits and the need for biomarker-informed combinations [26].

### 4.4. Limitations

Key caveats constrain interpretation. First, curated and literature-mined resources are biased toward well-studied genes; we reduced but cannot fully eliminate this bias by conservative cross-resource retention rules and provenance reporting. Second, network-proximity and transcriptomic correlations are associative; orthogonal validation (phospho-proteomics, perturbation screens) is required to establish causality. Third, cohort heterogeneity and variable sample sizes limit power to detect low-frequency events in rarer histologies; sensitivity analyses ameliorate but do not fully remove this limitation. Finally, transcriptomic module scores are proxies for phosphorylation-dependent mTOR activity—integrating phospho-proteomic datasets would strengthen causal claims and biomarker development [23,27].

### 4.5. Concrete Next Steps for Validation

We recommend immediate experimental priorities that follow from this pipeline: (i) targeted phospho-proteomic profiling of tumors and cell lines stratified by composite nomination (genotype + module activity); (ii) pooled CRISPR/RNAi perturbation of top network-proximal candidates in panels stratified by *PTEN/PIK3CA/MTOR* status; and (iii) retrospective testing of module-score predictivity in drug-sensitivity datasets (e.g., CCLE/PRISM) [28]. Successful retrospective validation should motivate prospective correlative cohorts in early-phase trials where composite biomarkers are measured alongside pharmacodynamic readouts (pS6, p-4E-BP1) [19].

## 5. Conclusions

The findings of this study indicate that the mTOR signaling pathway substantially contributes to cancer etiopathogenesis and represents a tractable target for therapeutic intervention. By integrating pathway membership, interaction topology, functional associations, disease aggregation, and protein-level variant context, we provide a coherent framework that links *MTOR*-centered network architecture to clinically actionable hypotheses. By explicitly linking genotype, topology and expression—and by mapping each link to compact, reproducible tests (H1–H3)—this revision converts a descriptive catalog of mTOR components into a falsifiable, mechanism-aware roadmap for biomarker nomination and experimental prioritization. We anticipate that the hypotheses and prioritized candidate lists produced here will accelerate targeted functional validation and enable more precise stratification in trials of rapalogs, next-generation mTOR kinase inhibitors, and combined PI3K/mTOR regimens. Future work should validate these hypotheses using phospho-proteomic readouts, perturbation assays, and stratified clinical designs that incorporate *PTEN/PIK3CA* status and *MTOR* variant information.

## Figures and Tables

**Table 1:** Variants of the *mTOR* gene associated with cancers.

Residue Change	dbSNP	Variant Description	Cancer Type
A8S, p.Ala8Ser	rs748801456	missense variant	lung large cell carcinoma
M2011V, p.Met2011Val	rs2100412651	missense variant	ovarian mucinous carcinoma
S2215Y, p.Ser2215Tyr	rs587777894	missense variant	colorectal adenocarcinoma
L2220F, p.Leu2220Phe	rs2100381099	missense variant	renal cell carcinoma
V2406A, p.Val2406Ala	rs2100316251	missense variant	renal cell carcinoma

**Table 2:** Disorders associated with mTOR signaling pathway SuperPath.

Disorder	Genes	Score
Follicular basal cell carcinoma	*TSC2*, *RICTOR*, *RHEB*, *RPS6KB1*, *MTOR*, *MLST8*, *TSC1*, *RPTOR*, *EIF4EBP1*, *AKT1*	11.84
Childhood ovarian dysgerminoma	*AKT1*, *EIF4EBP1*, *MLST8*, *TSC1*, *TSC2*, *RPS6KB1*, *RPTOR*, *MTOR*, *RHEB*, *RICTOR*	11.78
Paranoid schizophrenia	*AKT1*, *EIF4EBP1*, *TSC2*, *RPTOR*, *TSC1*, *MTOR*, *RHEB*, *MLST8*, *RPS6KB1*, *RICTOR*	11.78
Congenital lipomatous overgrowth, vascular malformations, and epidermal nevi	*MTOR*, *AKT1*, *TSC2*, *TSC1*	11.75
Tuberous sclerosis	*AKT1*, *EIF4EBP1*, *TSC1*, *RPS6KB1*, *TSC2*, *RHEB*, *MTOR*	11.70
Postauricular lymphadenitis	*AKT1*, *MAPK3*, *RAF1*	11.69
Mitochondrial complex iii deficiency, nuclear type 9	*AKT1*, *MTOR*, *TSC2*	11.68
Cowden syndrome 1	*AKT1*, *MTOR*, *TSC2*	11.65
Gastric adenocarcinoma	*AKT1*, *MTOR*, *RAF1*	11.64
Mitochondrial complex iv deficiency, nuclear type 7	*AKT1*, *MTOR*, *TSC2*	11.64

**Table 3:** Pan-cancer specificity metrics for mTOR-axis genes.

Gene	Mutated%_Pan	Amplified%_Pan	Entropy	Tau	Class	Top_Cancer	Second_Cancer	Top_vs_Second_Diff(%)	Chi2_p	BH-FDR_q
*PTEN*	9.681	7.803	0.786	0.881	Shared	ucec_tcga_pan_can_atlas_2018	gbm_tcga_pan_can_atlas_2018	10.568	0	0
*PIK3CA*	12.712	29.09	0.84	0.776	Shared	ucec_tcga_pan_can_atlas_2018	gbm_tcga_pan_can_atlas_2018	11.871	0	0
*RICTOR*	5.051	26.516	0.771	0.877	Shared	gbm_tcga_pan_can_atlas_2018	kirc_tcga_pan_can_atlas_2018	11.154	6.3 × 10^−230^	6.8 × 10^−230^
*RPS6KB1*	3.913	22.638	0.688	0.91	Shared	gbm_tcga_pan_can_atlas_2018	kirc_tcga_pan_can_atlas_2018	10.842	4 × 10^−304^	8 × 10^−304^
*MTOR*	6.12	9.812	0.815	0.846	Shared	gbm_tcga_pan_can_atlas_2018	kirc_tcga_pan_can_atlas_2018	6.414	9.7 × 10^−234^	1.2 × 10^−233^
*RPTOR*	4.816	23.649	0.766	0.885	Shared	gbm_tcga_pan_can_atlas_2018	kirc_tcga_pan_can_atlas_2018	11.713	5.8 × 10^−247^	8.8 × 10^−247^
*MLST8*	3.843	18.39	0.688	0.914	Shared	gbm_tcga_pan_can_atlas_2018	kirc_tcga_pan_can_atlas_2018	12.13	0	0
*AKT1*	4.146	13.953	0.72	0.904	Shared	gbm_tcga_pan_can_atlas_2018	kirc_tcga_pan_can_atlas_2018	11.597	2.6 × 10^−273^	4.5 × 10^−273^
*TSC1*	5.118	12.265	0.794	0.875	Shared	gbm_tcga_pan_can_atlas_2018	kirc_tcga_pan_can_atlas_2018	11.74	8.2 × 10^−244^	1.1 × 10^−243^
*TSC2*	5.402	18.357	0.817	0.866	Shared	gbm_tcga_pan_can_atlas_2018	kirc_tcga_pan_can_atlas_2018	11.349	2.6 × 10^−226^	2.6 × 10^−226^
*EIF4EBP1*	3.57	22.808	0.652	0.92	Shared	gbm_tcga_pan_can_atlas_2018	kirc_tcga_pan_can_atlas_2018	11.455	0	0
*RPS6*	3.839	10.057	0.682	0.912	Shared	gbm_tcga_pan_can_atlas_2018	kirc_tcga_pan_can_atlas_2018	11.455	1 × 10^−304^	2.5 × 10^−304^

Mutated% and Amplified% show study-level prevalences; Entropy and τ are normalized specificity metrics (0–1). Class = Shared/Tumor-specific/Ambiguous (cutoffs: Shared = ≥5% in ≥5 studies; Tumor-specific = top − second ≥10 percentage points). Gene-wise heterogeneity tested by χ^2^/Fisher; q = BH-FDR.

**Table 4:** Model summaries for the translational activity module across key mTOR-axis genotypes (pooled across cancer types).

Contrast	Module	Method	*p*	q (BH-FDR)	Cliff’s δ	Median Diff (1–0)	N0	N1	Median0	Median1	IQR0	IQR1
PTEN loss vs. WT	Translational	Mann–Whitney (Cliff’s δ)	0.073534146	0.110301219	−0.021835686	0.0286	6711	3360	−0.0335	−0.0049	0.72	0.7279
PIK3CA mut vs. WT	Translational	Mann–Whitney (Cliff’s δ)	0.054036275	0.110301219	0.032677061	−0.0304	8735	1336	−0.0214	−0.0518	0.7277	0.6772
RICTOR amp vs. diploid	Translational	Mann–Whitney (Cliff’s δ)	0.287450376	0.287450376	−0.013557931	0.0134	7201	2870	−0.0269	−0.0136	0.7075	0.753

**Table 5:** Top 10 non-mutated, network-proximal nominations.

Filtered_Rank	Gene_Symbol	Page_Rank_Score	Pan_Cancer_Alteration_Pct (%)	Degree	Source_Support_Count
1	*MAP2K1*	0.018697	0.5	184	3
2	*PIK3CA*	0.018113	2.1	19	5
3	*MAPKAP1*	0.017997	1.1	64	2
4	*AKT2*	0.017284	0.1	168	1
5	*TSC2*	0.015384	0.9	76	4
6	*GNB1*	0.014972	0.1	150	4
7	*PAX6*	0.014652	2.0	195	5
8	*SOS1*	0.013520	0.6	31	5
9	*PRAS40*	0.013374	1.4	170	1
10	*EIF4E*	0.013126	1.0	165	4

The top ten genes nominated by PageRank propagation that pass the non-mutated filter (pan-cancer alteration frequency ≤ 3%) and are not flagged for recurrent activating hotspots. Columns: filtered_rank (rank among filtered candidates), gene_symbol, page_rank_score (steady-state influence), pan_cancer_alteration_pct (%), degree (STRING degree), source_support_count (number of supporting sources).

## Data Availability

The original contributions presented in this study are included in the article/Supplementary Materials. Further inquiries can be directed to the corresponding author.

## Supplementary Materials

The following supporting information can be downloaded at: https://www.mdpi.com/article/10.3390/genes16111253/s1, Supplementary Figure S1: Analytical workflow and data integration.. Supplementary Table S1. List of genes associated with the mTOR Signaling Pathway via GeneCards. Supplementary Table S2. List of genes associated with mTOR-signaling MAP-165159. Supplementary Table S3. Pairwise mutual exclusivity and co-occurrence among mTOR-axis alterations. Odds ratios (OR) from 2 × 2 alteration matrices summarize dependence between gene pairs across tumors.
