# Supplementary material for: Bioinformatics Approach to mTOR Signaling Pathway-Associated Genes and Cancer Etiopathogenesis
Source: Genes (Basel). 2025 Oct 24;16(11):1253. doi: 10.3390/genes16111253 (PMC12652958; doi:10.3390/genes16111253)
Supplement: Supplementary file 1 [file genes-16-01253-s001.zip › Supplementary Table S1.pdf]

Supplementary Table S1. List of genes associated with the mTOR Signaling Pathway via GeneCards

**Gene Symbol**	**Gene full name**	**Relevance Score (RS)**
MTOR	Mechanistic Target Of Rapamycin Kinase	191.72
AKT1	AKT Serine/Threonine Kinase 1	104.60
MAPK1	Mitogen-Activated Protein Kinase 1	92.77
PIK3CA	Phosphatidylinositol-4,5-Bisphosphate 3-Kinase Catalytic Subunit Alpha	90.67
STAT3	Signal Transducer And Activator Of Transcription 3	87.65
EGFR	Epidermal Growth Factor Receptor	76.79
CTNNB1	Catenin Beta 1	75.64
MAPK3	Mitogen-Activated Protein Kinase 3	70.29
PTEN	Phosphatase And Tensin Homolog	69.71
TP53	Tumor Protein P53	65.99
STAT1	Signal Transducer And Activator Of Transcription 1	64.73
RPTOR	Regulatory Associated Protein Of MTOR Complex 1	64.73
MAPK8	Mitogen-Activated Protein Kinase 8	61.73
MAPK14	Mitogen-Activated Protein Kinase 14	61.23
RAF1	Raf-1 Proto-Oncogene, Serine/Threonine Kinase	59.97
MAP2K1	Mitogen-Activated Protein Kinase Kinase 1	59.15
PIK3CG	Phosphatidylinositol-4,5-Bisphosphate 3-Kinase Catalytic Subunit Gamma	59.01
PIK3R1	Phosphoinositide-3-Kinase Regulatory Subunit 1	58.13
BRAF	B-Raf Proto-Oncogene, Serine/Threonine Kinase	58.03
APC	APC Regulator Of WNT Signaling Pathway	57.87
RPS6KB1	Ribosomal Protein S6 Kinase B1	57.46
TGFB1	Transforming Growth Factor Beta 1	57.39
SRC	SRC Proto-Oncogene, Non-Receptor Tyrosine Kinase	57.16
NFKB1	Nuclear Factor Kappa B Subunit 1	56.97
TNF	Tumor Necrosis Factor	56.75
PTPN11	Protein Tyrosine Phosphatase Non-Receptor Type 11	56.29
KRAS	KRAS Proto-Oncogene, GTPase	55.37
JAK2	Janus Kinase 2	54.87
IL6	Interleukin 6	53.34
MYD88	MYD88 Innate Immune Signal Transduction Adaptor	53.28
RICTOR	RPTOR Independent Companion Of MTOR Complex 2	52.60
HRAS	HRas Proto-Oncogene, GTPase	52.04
VEGFA	Vascular Endothelial Growth Factor A	51.91
GSK3B	Glycogen Synthase Kinase 3 Beta	51.81
GRB2	Growth Factor Receptor Bound Protein 2	51.57
JUN	Jun Proto-Oncogene, AP-1 Transcription Factor Subunit	51.19
BDNF-AS	BDNF Antisense RNA	50.39
MLST8	MTOR Associated Protein, LST8 Homolog	50.15
