# Supplementary material for: Bioinformatics Approach to mTOR Signaling Pathway-Associated Genes and Cancer Etiopathogenesis
Source: Genes (Basel). 2025 Oct 24;16(11):1253. doi: 10.3390/genes16111253 (PMC12652958; doi:10.3390/genes16111253)
Supplement: Supplementary file 1 [file genes-16-01253-s001.zip › Supplementary Table S2.pdf]

Supplementary Table S2. List of genes associated with mTOR-signaling MAP-165159

**Gene symbol**	**Gene full name**
STRADB	STE20-related kinase adapter protein beta
TSC2	Tuberin
RPS6KB1	Ribosomal protein S6 kinase beta-1
PRKAB2	5'-AMP-activated protein kinase subunit beta-2
LAMTOR5	Ragulator complex protein LAMTOR5
CAB39	Calcium-binding protein 39
RHEB	GTP-binding protein Rheb
RRAGB	Ras-related GTP-binding protein B
EEF2K	Eukaryotic elongation factor 2 kinase
LAMTOR1	Ragulator complex protein LAMTOR1
PRKAG2	5'-AMP-activated protein kinase subunit gamma-2
TSC1	Hamartin
RPTOR	Regulatory-associated protein of mTOR
PRKAG1	5'-AMP-activated protein kinase subunit gamma-1
STK11	Serine/threonine-protein kinase STK11
PPM1A	Protein phosphatase 1A
STRADA	STE20-related kinase adapter protein alpha
EIF4EBP1	Eukaryotic translation initiation factor 4E-binding protein 1
LAMTOR4	Ragulator complex protein LAMTOR4
PRKAA1	5'-AMP-activated protein kinase catalytic subunit alpha-1
MTOR	Serine/threonine-protein kinase mTOR
LAMTOR2	Ragulator complex protein LAMTOR2
RRAGD	Ras-related GTP-binding protein D
PRKAA2	5'-AMP-activated protein kinase catalytic subunit alpha-2
YWHAB	14-3-3 protein beta/alpha
RRAGC	Ras-related GTP-binding protein C
RPS6	40S ribosomal protein S6;
RRAGA	Ras-related GTP-binding protein A
AKT1S1	Proline-rich AKT1 substrate 1
AKT2	RAC-beta serine/threonine-protein kinase
SLC38A9	Sodium-coupled neutral amino acid transporter 9
FKBP1A	Peptidyl-prolyl cis-trans isomerase FKBP1A
EIF4B	Eukaryotic translation initiation factor 4B
PRKAG3	5'-AMP-activated protein kinase subunit gamma-3
EIF4G1	Eukaryotic translation initiation factor 4 gamma 1
LAMTOR3	Ragulator complex protein LAMTOR3
EIF4E	Eukaryotic translation initiation factor 4E;
PRKAB1	5'-AMP-activated protein kinase subunit beta-1
AKT1	RAC-alpha serine/threonine-protein kinase
MLST8	Target of rapamycin complex subunit LST8
CAB39L	Calcium-binding protein 39-like
