# Supplementary material for: Bioinformatics Approach to mTOR Signaling Pathway-Associated Genes and Cancer Etiopathogenesis
Source: Genes (Basel). 2025 Oct 24;16(11):1253. doi: 10.3390/genes16111253 (PMC12652958; doi:10.3390/genes16111253)
Supplement: Supplementary file 1 [file genes-16-01253-s001.zip › Supplementary Table S3.pdf]

Supplementary Table S3. Pairwise mutual exclusivity and co-occurrence among mTOR-axis alterations. Odds ratios (OR) from 2 × 2 alteration matrices summarize dependence between gene pairs across tumors.

Gene A	Gene B	Odds ratio	Chi2 (Yates)	p	a (11)	b (10)	c (01)	d (00)	q_BH
PIK3CA	*RICTOR*	3.356418407	0.293802047	0.587794	1936	2409	1279	5343	0.877003
PIK3CA	*RPS6KB1*	2.416378029	0.141956802	0.706343	1492	2853	1178	5444	0.877003
PIK3CA	*MTOR*	2.720191087	0.117673004	0.731572	908	3437	586	6036	0.877003
PIK3CA	*PTEN*	1.899331359	0.091252011	0.762591	1923	2422	1952	4670	0.877003
PIK3CA	*TSC1*	2.045975216	0.09529333	0.757553	1448	2897	1300	5322	0.877003
PIK3CA	*TSC2*	2.744535906	0.147243011	0.701184	1158	3187	774	5848	0.877003
RICTOR	*RPS6KB1*	2.942894331	0.203944028	0.651556	1267	1948	1403	6349	0.877003
RICTOR	*MTOR*	2.643719559	0.111981153	0.7379	725	2490	769	6983	0.877003
RICTOR	*PTEN*	1.575723514	0.040640412	0.840234	1377	1838	2498	5254	0.877003
RICTOR	*TSC1*	2.577286025	0.157970531	0.691032	1236	1979	1512	6240	0.877003
RICTOR	*TSC2*	2.815336182	0.153966398	0.694773	940	2275	992	6760	0.877003
RPS6KB1	*MTOR*	1.80606518	0.036285456	0.848927	518	2152	976	7321	0.877003
RPS6KB1	*PTEN*	1.447564146	0.023953475	0.877003	1118	1552	2757	5540	0.877003
RPS6KB1	*TSC1*	2.217519942	0.101861938	0.749607	995	1675	1753	6544	0.877003
RPS6KB1	*TSC2*	2.026181071	0.064188117	0.799995	698	1972	1234	7063	0.877003
MTOR	*PTEN*	2.272360135	0.08019462	0.777034	783	711	3092	6381	0.877003
MTOR	*TSC1*	2.618398211	0.105813347	0.744962	640	854	2108	7365	0.877003
MTOR	*TSC2*	2.718753995	0.099739827	0.752142	490	1004	1442	8031	0.877003
PTEN	*TSC1*	1.987707575	0.086230268	0.769025	1305	2570	1443	5649	0.877003
PTEN	*TSC2*	2.194330887	0.089577505	0.764715	982	2893	950	6142	0.877003
TSC1	*TSC2*	3.087388654	0.177510367	0.673522	866	1882	1066	7153	0.877003
