# Supplementary figures and images for: Bioinformatics Approach to mTOR Signaling Pathway-Associated Genes and Cancer Etiopathogenesis
Source: Genes (Basel). 2025 Oct 24;16(11):1253. doi: 10.3390/genes16111253 (PMC12652958; doi:10.3390/genes16111253)

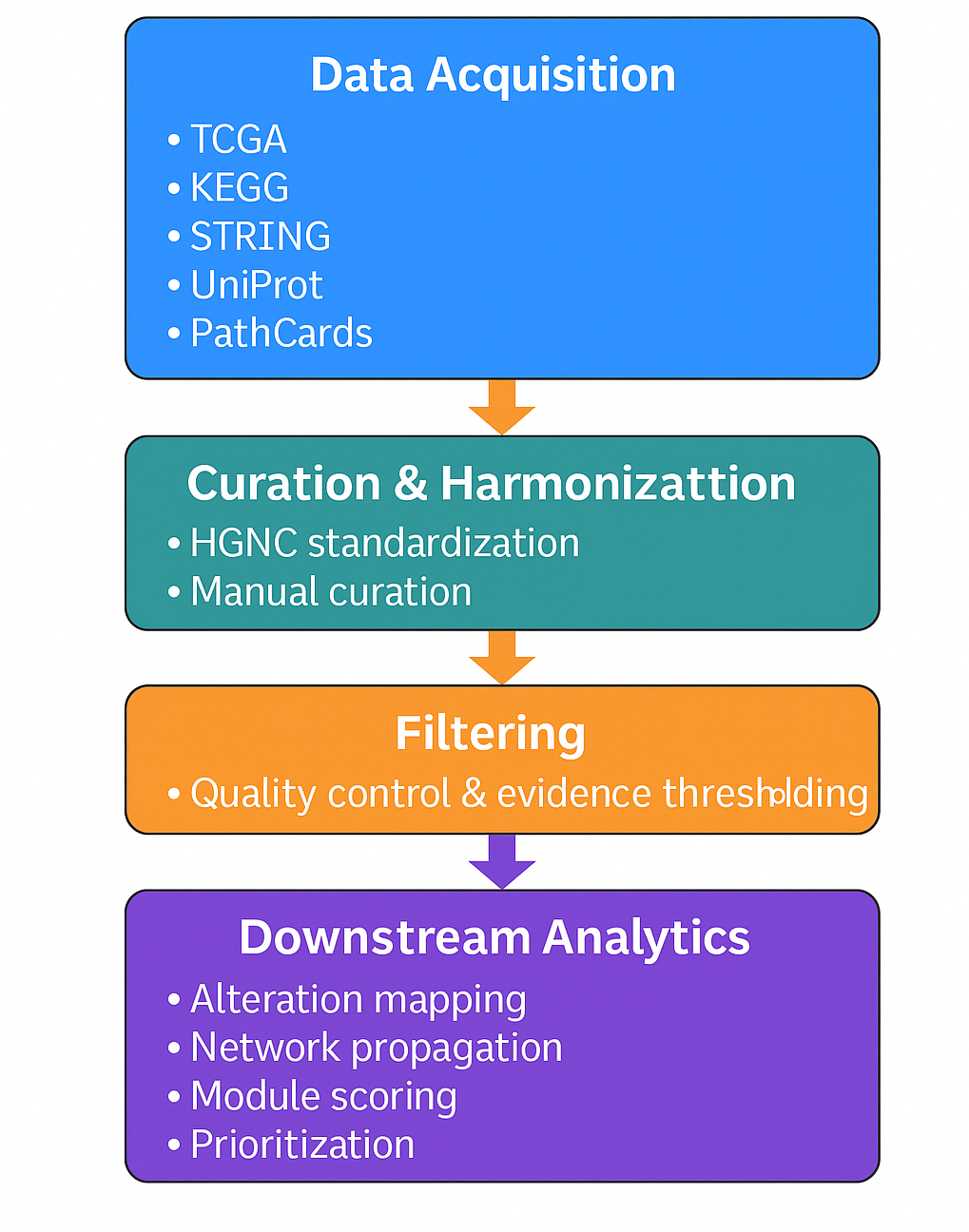

Supplement: Supplementary file 1 [file genes-16-01253-s001.zip › Supplementary Figure S1.png]
